# A 10-Year Review of Sunburn Injuries Presenting to the Manchester Adult and Paediatric Specialist Burn Services

**DOI:** 10.3390/ebj3040041

**Published:** 2022-09-29

**Authors:** Lewis A. Dingle, Poh Tan, Parisha Malik, Samantha McNally

**Affiliations:** 1 Burns and Paediatric Plastic Surgery Service, Royal Manchester Children’s Hospital, Oxford Road, Manchester M13 9WL, UK; 2 Blond McIndoe Laboratories, Centre for Tissue Injury and Repair, Faculty of Biological, Medical and Human Sciences, Stopford Building, Oxford Road, Manchester M13 9PT, UK; 3 Wythenshawe Burns Centre, Wythenshawe Hospital, Southmoor Road, Wythenshawe, Manchester M23 9LT, UK

**Keywords:** burn, sunburn, paediatric, adult

## Abstract

Background: The incidence of sunburn injuries continues to rise despite increased awareness of the risks of sun exposure and availability of sun protection. Whilst not a significant burden on burns care services, patients remain exposed to associated risks for future development of skin malignancies. The aims of this study were to determine the burden and severity of sunburn injury presentations to the Manchester adult and children’s burns services. Methods: A 10-year retrospective review was performed of patients with sunburn injuries, presenting to the Manchester burn services between 2010 and 2019 (inclusive). Data were collected from the International Burn Injury Database (iBID), electronic patient record (EPR) and local data collection systems. The data extracted included patient demographics, sunburn characteristics and management of the burn injury including need for admission and any documented surgical interventions. Temporal correlation was determined by linear regression analysis. Results: In total, 131 paediatric and 228 adult patients with sunburn injuries were managed by the Manchester burns services over the 10-year period. Mean % total body surface area burned was low (2.00% and 2.12% in adult and paediatric patients, respectively), with the majority of injuries either superficial or superficial partial thickness. Thirty percent (30.2%) of adult and 40.5% of paediatric patients were admitted with a mean length of stay of 3.51 and 1.11 days, respectively. The presentation of sunburn injuries progressively increased over the study period with a peak in 2017 (*n* = 58). Similar trends in patient demographics, burn size and depth and temporal trends were observed in national data from the same period for both adult and paediatric patients. Conclusion: This 10-year retrospective cohort study demonstrates an increasing trend of sunburn injury presentations to the Manchester specialist burns services; a pattern replicated in national data from England and Wales. The majority of sunburn injuries do not present to specialist burn services; therefore, these reported injuries reflect only a fraction of the true burden of sunburn nationwide. Despite increased awareness, an obvious need for enhanced public awareness campaigns regarding sun protection is therefore needed to address this trend. The educational and preventative role of burns care services is a key component in tackling both consequences of burn injuries themselves and associated risks such as future skin cancer development.

## 1. Introduction

The adult and paediatric burn services at Wythenshawe Hospital and Royal Manchester Children’s Hospital (RMCH) function as the major regional burn centres for the Greater Manchester area, serving a population of approximately 3 million people [1]. The adult burn service sees approximately 250 inpatient admissions and 600 new burn outpatients each year, whilst the paediatric service at RMCH manages ~800 inpatients (including non-overnight ‘ward attenders’) and ~500 outpatients. Although representing a relatively small proportion of burns, sunburn injuries are regularly referred to both services. Whilst the majority of these are treated in the outpatient setting or as ‘ward attenders’, some patients require admission for debridement, dressing management or analgesia. One expects such injuries to occur domestically during the Northern Hemisphere summer months. However, we regularly see sunburn injuries in patients returning from overseas travel due to our proximity to Manchester airport, a major international transport hub. Anecdotally, such injuries appear to be increasing at both our adult and paediatric centres.

This observation is supported from articles published in the media highlighting an increase in sunburn injuries in UK residents. Multiple charitable, academic and scientific bodies have warned of the high numbers of British patients suffering sunburn injuries, at a rate which appears to be increasing annually. In 2010 Cancer Research UK reported that up to 50% of British residents reported an episode of sunburn that summer [2], whilst in 2015 the British Association of Dermatologists (BAD) noted this had increased further with 72% reporting a sunburn episode in the previous year [3]. More recent surveys suggest at least one-third of Britons admit to experiencing one episode of sunburn each year [4,5], with significant numbers being burnt domestically in the UK [6,7]. Evidence suggests this is also not a purely British phenomenon with high sunburn incidence reported in other sun-seeking populations [8].

The burden of these injuries acutely and on burn care services is not the only reason for concern regarding excessive sun exposure. Rates of skin cancer are doubling every 10–20 years, with nearly 20,000 new melanoma cases per year noted in the UK [9] alongside over 200,000 non-melanoma skin cancers (NMSC) [10]. This now equates to a 1 in 5 lifetime risk for developing skin cancer. This rise likely reflects the availability of low-cost travel to sun-rich countries from the 1970s onwards, coupled with the rise of indoor tanning beds in the 1980s through the 2000s. Furthermore, an increasingly ageing population allows for increased cumulative sun exposure over a lifetime; a suggested risk factor for development of NMSC [11]. Increased melanoma risk conversely is associated with excessive high intensity sun exposure as a result of discrete episodes of burning [12]. Consequently, over 80% of melanoma skin cancer cases are thought to be preventable, simply through better sun protection and modifications in behaviour [13].

Such injuries continue to occur despite widespread high awareness (~90%) of appropriate sun protection behaviour [4,6] and availability of sunscreen agents. More concerning, given their early exposure to burning, paediatric sunburn injuries also appear to be increasing. This is suggested, in part, to be due to lack of parental concern regarding sun exposure [14], poor use of sunscreen and lack of awareness of good sun protective behaviour [15].

Based on our experience of increasing sunburn cases presenting locally in Manchester, we wished to ascertain the burden of sunburn injuries on our service and identify any trends in presentations over time. We also wished to compare this to national trends in data recorded for England and Wales in the International Burn Injury Database (iBID).

## 2. Materials and Methods

A 10-year retrospective review of adult and paediatric patients with sunburn injuries presenting to the Manchester Adult Burns Centre at Wythenshawe Hospital and RMCH between 2009 and 2019 (inclusive) was performed. Patients were identified using the International Burn Injury Database (iBID), searching for ‘sunburn’ or ‘radiation’ as the mechanism of injury. Screening of individual results was performed to ensure accurately recorded mechanism of injury. Patients with alternative mechanisms of injury such as sunbed injuries or other burn aetiologies were excluded. Information within the iBID entry, the electronic patient record (EPR), our local telemedicine referral system (Medical Data Solutions and Services (MDSAS), Manchester, UK) and local data collection systems were used to obtain data. Data extracted from these resources included patient demographics, characteristics of the sunburn injury, geographical location of injury, admission information and management details, where available. Where a patient was documented as an admission, the minimum length of stay (LOS) was rounded to one day, to reflect bed occupancy. For all admissions greater than one day, LOS was rounded to the nearest whole integer.

Additional data covering the same 10-year period was obtained by request from the iBID database for England and Wales as a comparison. Only information uploaded to iBID was available for national patients. Individual patient screening was not performed for national data.

Data was analysed using Microsoft^®^ Excel (2022 v16.59, Office365, Microsoft^®^) and GraphPad Prism 9 for macOS (Version 9.3.1 (350), 7 December 2021, GraphPad Software).

## 3. Results

### 3.1. Patient Demographics and Sunburn Characteristics

**Figure 1:**
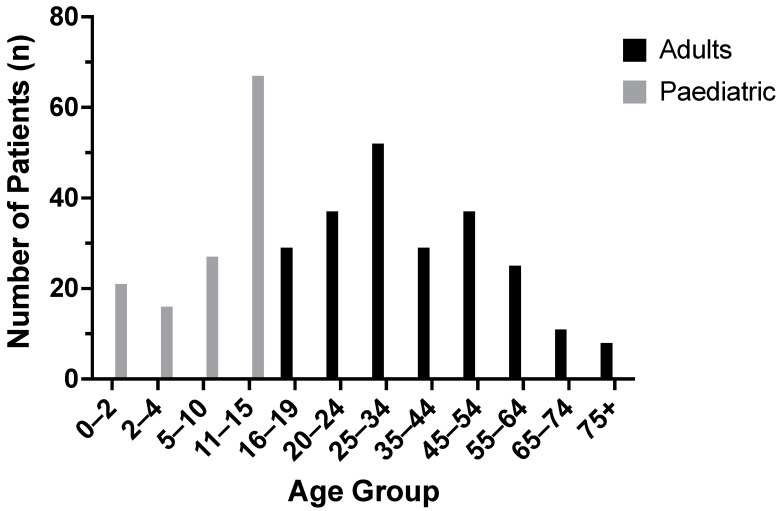
Age of patients presenting to the combined Manchester adult and paediatric burn centres between 2010–2019 (inclusive).

In total, 228 adult patients with a diagnosis of sunburn injury were treated by the Manchester burn service during the 10-year period (2010–2019 inclusive). The mean age of adult patients was 38 years (Range 16–83), in keeping with the typical sunseeker likely to sustain a sunburn injury. An equal number of male and female patients were affected (Table 1). When stratified by age, 52% of adult sunburn injuries occurred in patients between 16 and 35 years of age (Figure 1). Over the same period, 131 paediatric patients were identified with sunburn injuries. The majority of paediatric patients were aged between 5–15 years (94, 72%) whilst 21 patients were under 1 year of age (16%, Figure 1).

Mean total body surface area (TBSA) burned was low in both adult (2.00%) and paediatric (2.12%) patients (Table 1), as expected. Seven adult patients sustained burns greater than 10% TBSA, with 14% TBSA the single largest recorded injury, whereas no paediatric burns were greater than 10%.

The majority of burn injuries were either superficial or superficial partial thickness (SPT), with only 5% (11/228) of adult patients presenting with deeper injuries. In paediatric patients only a single patient presented with deeper injuries (1/131, 0.8%). Surprisingly, 11 adult patients (5%) were conveyed to the Manchester burns unit via ambulance transfer despite a mean age of 44 years and mean TBSA of only 3.25%. Only one of these patients had an injury greater than 10% TBSA on assessment at the burn centre. No adult or paediatric patients required formal resuscitation, surgical intervention, nor were there any mortalities.

**Figure 2:**
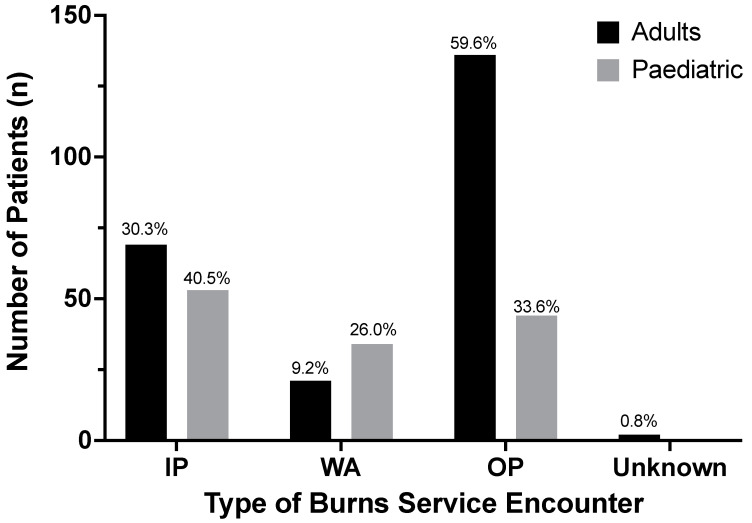
Management of patients with sunburn injury admitted to Manchester adult and paediatric burn centres between 2010–2019 (inclusive). % of cohort shown above bar (*n* = 288 (adults), 131 (paediatric)).

**Figure 3:**
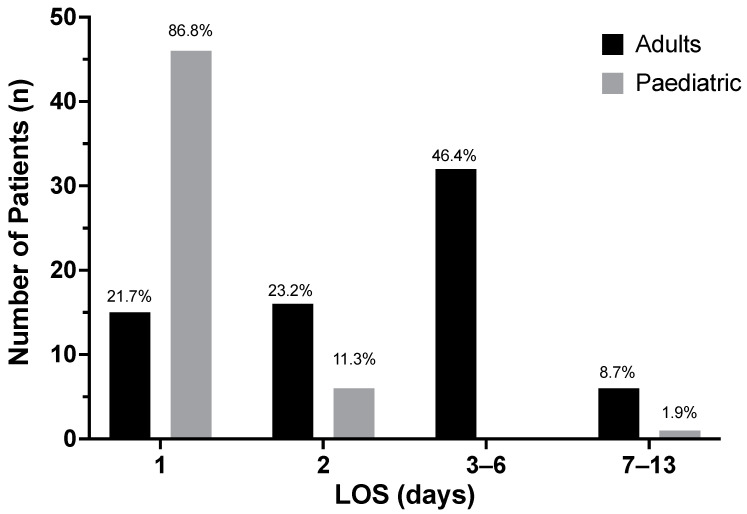
Length of Stay (LOS) in days of adult and paediatric patients admitted with sunburn injuries to the Manchester burn centres between 2010–2019 (inclusive).

In total, 30.2% of adult patients (69/228) were documented as requiring admission to the burn centre, with the remaining patients managed either in the outpatient setting, or as a ‘ward attender’ (burn centre ward attendance but without planning/intention for formal admission) (Figure 2). In the paediatric cohort 40.5% (53/131) patients were admitted, whilst 59.5% (78/131) were managed as either outpatients or as ‘ward attenders’ (less than 12 h non-formal admission to the ward, Figure 2). ‘Ward attenders’ were documented separately on iBID allowing for their identification. In patients admitted, the mean LOS was 3.51 days (SD 2.42, range 1–13) for adults and 1.11 days (SD 1.27, range 1–10) for children. In paediatric patients, 86.8% (46/53) required an admission of one day, 11.3% (6/53) two days, whilst only a single patient required over 7 days (Figure 3).

### 3.2. Chronological Trend in Sunburn Injuries

**Figure 4:**
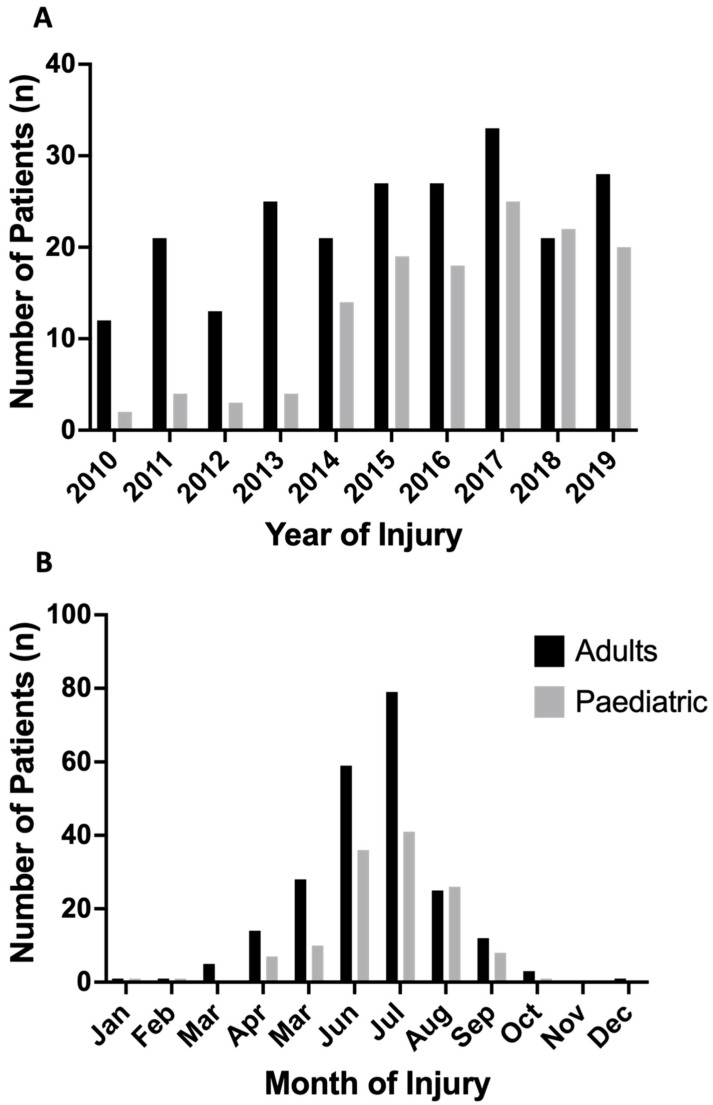
Adult and paediatric sunburn injuries stratified by date of injury. (**A**) sunburn injuries per year, demonstrating clear trend of increasing injury over study period. * note—limited record keeping in paediatric data prior to 2016 may exacerbate trend due to artificial low numbers in years prior to 2016. (**B**) sunburn injuries by month between 2010–2019, demonstrating predominance of injuries during northern hemisphere summer months (May–August) in adult and paediatric patients.

Over the 10-year period, an increasing trend in cases was observed, with a low of 12 adult cases in 2010, compared to 33 and 28 in 2017 and 2019, respectively (Figure 4A). This was mirrored in the paediatric data with an upward trend of sunburn patients from 2010 (*n* = 2) to 2019 (*n* = 20), with a peak in 2017 (*n* = 25).

Weekends accounted for over one third of sunburn injuries (adults—86/228, 37.7%, paediatric—43/131, 32.8%). Approximately 89.9% (205/228) and 86.3% (113/131) of adult and paediatric sunburn injuries, respectively, occurred in the summer months (May to August), with a peak of 79 adult and 41 paediatric patients in July (Figure 4B).

### 3.3. Geographical Location

**Figure 5:**
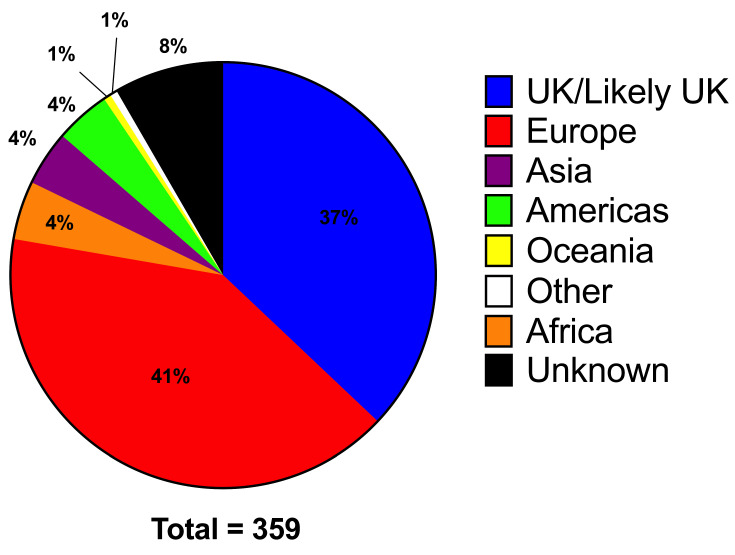
Geographical location of sunburn injury in combined adult and paediatric patients between 2010–2019 (inclusive). *n* = 359 (288 adults, 131 paediatric).

Location of injury was poorly recorded in iBID data. Where possible, location of injury in adult patients was confirmed by examining both iBID free text information, MDSAS referral information and the EPR. Location was not clearly described/documented in 53 (23.2%) of adult sunburn injuries. Domestic injuries were the most common single geographical location for paediatric (48.1%, 63/131) and second most common for adult (18%, 43/228) sunburn injuries. In adult patients, a further 27 (11.8%) injuries were likely to have occurred in the UK based on available information (such as description, date of injury vs. presentation). When combined, injuries occurring overseas accounted for over half of all cases (196/359 54.6%), with location undocumented in 30 cases. In keeping with UK holiday trends, the most common overseas country in which burn injuries occurred was Spain (25.4% adult, 26.0% paediatric), however eastern Mediterranean countries such as Turkey, Greece, Cyprus and Egypt also accounted for a significant number of cases (Figure 5).

### 3.4. National Data

#### 3.4.1. Adults

A corresponding search of the iBID database for sunburn injuries across the UK over the same period (2010–2019) identified 1162 patients. Mean age of patient (36.8 years), male to female ratio (1.25:1) and % TBSA burn (2.3%, range 0–80%) were similar to the Manchester burn service cohort. By contrast, 13 sunburn injuries nationally required formal resuscitation (1.1%), with a single mortality (8% TBSA). In total, 17.6% of patients (204/1162) required hospital admission (documented as ‘admission’ on iBID), with a mean LOS of 3.5 days (Range 1–44, Median 2 days). Ten patients (0.83%) required formal surgical theatre visits, with six patients requiring skin grafting.

**Figure 6:**
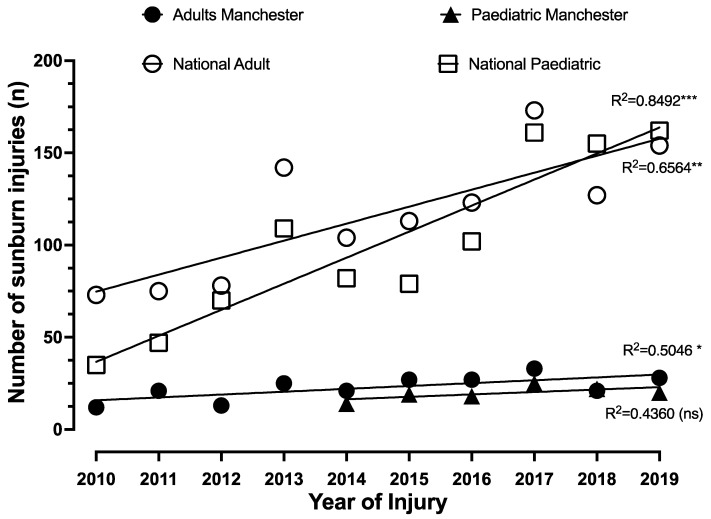
Temporal pattern in sunburn injuries comparing local (Greater Manchester) and National data from iBID for adult and paediatric patients demonstrating increasing trend over time (R^2^ value of linear correlation, *** *p* < 0.0005, ** *p* < 0.005, * *p* < 0.05).

Like the Manchester data, a trend in increasing sunburn presentations was noted across the study period, with 73 patients documented in 2010 compared to 173 in 2017 and 154 in 2019 (Figure 6). May, June, July and August again accounted for >83% of cases (14.3%, 25.6%, 33.6% and 9.8%, respectively).

#### 3.4.2. Paediatric

A comparable iBID database search across the UK over the same period identified 1002 paediatric patients. National demographics were similar to the Manchester cohort: male to female ratio was 2.3:1, % TBSA burn was 2.3% (Range 0–51%) and the mean age of patients was found to be 7 years, with 20% (203/1002) of the patients being in the age group of 0–1 years. Unlike adults, no formal resuscitation or mortality was reported in the paediatric population nationally, despite documented TBSA >10% in 22 patients. This may reflect limitations of iBID documentation. Hospital admission was required in 25.7% of paediatric patients (258/1002), with a mean LOS of 1.5 days (Range 1–13, Median 1 day). Thirteen patients required surgical management in theatres (1.3%), with six patients requiring skin grafting.

An increase in sunburn presentations across the study period was also noted in this population, with 35 patients documented in 2010 compared to 162 in 2019 (Figure 6), which was similarly reflected in our Manchester data. The summer months again accounted for > 91% of cases (13.4%, 30.6%, 36.6%, 11.0%, respectively).

Due to noted limitations in iBID recording of ‘location of injury’ from the local Manchester data, national data was not deemed reliable for analysis and comparison to local data.

## 4. Discussion

This 10-year retrospective study of patients with sunburn injuries managed by burns centres in Manchester demonstrates that whilst not a significant burden in terms of case load (35 cases per year on average combined) these easily preventable burn injuries account for a proportion of specialist burn centre cost and time. In addition, a small but reasonable number of patients each year require admission, occupying a precious resource and compounding the national shortage of specialist burns beds in the region and England and Wales more broadly. Although most injuries are small, occasional major burn injuries result from sun exposure that requires formal resuscitation and surgical intervention in England and Wales.

The temporal pattern observed in our study may be due to a number of factors. Firstly, increased referrals to specialist burn services in an increasing population size would account for greater numbers of apparent sunburn injury. Indeed, the UK population has increased by an estimated 5 million persons during the study period [1]. Alternatively, well documented increases in global temperature resulting from climate change may lead to increased opportunities for sun exposure in countries such as the UK. Interestingly, however, 2017, the year with the most sunburn injuries treated at burn centres in our study, had neither the highest average daily temperature or hours of sunshine either in the summer or annually [16].

Other database studies support the findings noted in our study. A recent review of the BRANZ (Burns repository of Australia and New Zealand) database by Connolly et al. found that sunburn accounted for 0.6% of burn injury admissions between 2010–2019, equating to 167 admissions [17]. As in our study, the majority of these were superficial, however unlike in our cohort, nearly 20% of patients required surgical management and 4% required intensive care admission. This highlights that despite UK data suggesting that the majority of sunburn injuries are trivial or uncomplicated, such injuries can be life-threatening. Connolly et al. did not comment on the temporal pattern of injuries over the 10-year study period, although concluded there was a need for further ANZBA (Australia and New Zealand Burns Association) campaigns and educational endeavours to improve sunburn awareness.

Whilst absolute case numbers are low, we have found an increasing trend of sunburn injuries in both adults and children despite widespread availability of both sun protection and increased education of the long-term risks of excessive sun exposure. This suggests that, despite increased awareness, young patients continue to suffer sunburn injuries and thus may be accumulating significant cumulative sun exposure and/or experiencing burning episodes throughout life. Analysis of UK melanoma cancer data by Memon et al. identifies a lifetime incidence of 1 in 75 for development of malignant melanoma, which has increased by approximately over 700% in men and 300% in women since 1980 [18]. Alongisde this rise in melanoma cases, an estimated 400,000 new NSMC cases are predicted by 2025 [19]. Deaths from malignant melanoma have increased two-fold since 1970 to over 2300 deaths per year [20] and are projected to rise further to over 3000 deaths per year by 2035 [21]. Melanoma now represents the 5th most common cause of cancer and one of the top 20 causes of cancer death. An additional 1000 deaths per year result from NMSC [22]. Much of this increase can be attributed to the rise in ‘high intensity recreational UV exposure’ such as that encountered during sunbathing or use of tanning beds. Similar trends have been observed in large US cohort studies [23].

Exposure to UV radiation for younger patients is particularly worrying. Evidence suggests that early childhood and adolescent exposure to ultraviolet (UV) radiation is a significant risk factor for future development of melanoma [24,25,26], though whether this is through cumulative exposure or intermittent intense exposure remains uncertain [25]. Despite recent data suggesting a plateauing of melanoma development in younger patients over the last twenty years, early exposure may contribute to the ongoing rise in melanoma observed in older cohorts of patients [18].

Similar studies in other countries suggest sunburn injury is also a significant problem. One might expect increased awareness and practice of good sun protection behaviour in countries with more consistent sunshine prevalence. However, de Troya Martín et al. surveyed over 1000 ‘beachgoers’ in the Costa del Sol (Spain) and found almost half of respondents reported being burned at least once in the previous year [8], despite over 90% of respondents reporting that sun exposure was the major cause of skin cancer. In a study of 512 paediatric patients in Brazil, Andreola et al. noted that 96% of respondents were aware of the risk of skin cancer, however 69% reported an episode of sunburn in the previous 12 months [27]. In total, 27% of respondents had reported three or more episodes of sunburn and 10% admitted to not using sun protection at all. Similar prevalence of previous sunburn episodes has been reported in numerous surveys across the developed world, in paediatric, adolescent, and adult populations [28,29,30,31,32].

Importantly, cases presenting to burns services represent only the tip of the iceberg with regard to overall sunburn injuries in the UK. Over 93 million overseas visits were made by UK travellers in 2019, the majority of which were to Spain and other European countries [33]. A further 60 million domestic vacations and 120 million ‘day-trips’ were taken in 2019 (the last year before the impact of COVID-19 on the leisure and tourism industry) [34]. These figures have shown a continual upward trend. Our Manchester data suggests that domestic exposure is the most common geographical location for paediatric sunburn injuries (48%) and the second most common for adults (up to 30%). Overall, however, overseas burn injuries account for just over half of sunburn injuries (Figure 5) Whilst patients may be more likely to recognise the risk of overseas sunburn injury in countries with sunnier climes [5], our local data indicates that equal sun protection awareness is required in the UK. Indeed, recent data from Cancer Research UK suggests a third of UK adults do not routinely protect their skin from sun exposure in the UK, with 3.7 million adults admitting to using no protection even when the sun is strong [35]. This reinforces the need to continue efforts to improve public awareness of sun protection particularly in the summer months domestically. Increasing overseas sunburn injuries may reflect the increase in tourism numbers over the study period, rather than a declining awareness or practicing of sun protection when on holiday. More robust data collection would be required to extrapolate to the national data, or to identify whether an increase in domestic or overseas sunburn injuries in particular account for the rising trend in presentations.

Burns units can play a role in reinforcing this essential message, not only to prevent consequences of burn injury itself but to tackle the larger issue of rising skin cancer cases. An example of this is the targeted campaign in England and Wales focussing on scald injuries (Safe Tea Campaign) has proven successful in reaching parents and patients at risk of such injuries [36]. Both the British Dermatology Association and Cancer Research UK both promote a ‘Sun Awareness’ week in the United Kingdom [37], in an attempt to educate the public on risks of sun exposure, however this study demonstrates that injuries are continuing to occur at an apparently increasing incidence. Inclusion of the burns community, with expertise in national campaigns such as SafeTea and that National Burns Awareness Days, may help to improve the impact of such public awareness campaigns. The need for such campaigns remains, with evidence suggesting that despite the increased awareness, parents in particular remain unconcerned about the impact of sun exposure on children [18].

Additional information on the correct usage of sunscreen may also be of helpful, with reminders of the expected duration of benefit, recommendations for application and storage [38,39] all potentially reducing incidence of sunburn injuries. The Australian ‘Slip, Slap, Slop’ and ‘SunSmart’ campaigns provides an example of how such a campaign can be successfully implemented. First introduced 40 years ago in 1980 (Slip, Slop, Slap), with SunSmart following in 1988, they are credited with the significant reduction in skin cancer incidence in Australia [40,41]. Cost-effectiveness studies indicate they have been an efficient approach to reducing skin cancer incidence, however there is acknowledgement that more needs to be done to reduce incidence further [42]. Systematic reviews of interventions to reduce skin cancer [43] and interventions specifically targeted at adolescents suggest such campaigns can be effective and should make use of technology to increase engagement [44]. Understanding of the psychology underpinning sun exposure and protection, may facilitate more effective intervention design [27,29,45,46,47]. This includes educating and changing behaviour of parents, with whom responsibility lies for preventing excessive sun exposure in the youngest patients as well as children themselves who have been shown to clearly understand sun exposure risks and engage with targeted interventions in numerous settings [32,48,49,50,51].

Whilst this study focussed on sunburn injury from naturally occurring ultraviolet light, additional risk is present in the use of sunbeds or tanning devices which are commercially available. Latest data suggests up to 2.5% of 15–17-year-olds continue to use commercial sunbeds in the UK despite a 2011 ban on their use by customers under the age of 18 (a feature observed across the majority of Western Europe) [47,52]. Some countries, such as Australia, have gone further with outright bans on sunbeds for all ages [49]. Cost-effective interventions to reduce sunbed usage in the UK predict significant reductions in future skin malignancy [53] and should be considered alongside educational campaigns to reduce ultraviolet radiation exposure from natural sources. This would reinforce the message, that although there are noted benefits from sunlight exposure on vitamin D production, general health and mental well-being [54,55,56,57], all UV light exposure contains risk and education on appropriate precautions should be widely promoted.

### Limitations

There are a number of limitations to this study. We acknowledge that patient identification was dependent upon accurate data input to iBID. Such information may have been incomplete resulting in patients both locally in Greater Manchester and across the UK having been omitted. Secondly, accuracy of information in iBID is dependent on the data provided to, and entered by, the iBID data-entry personnel, which may therefore contain errors. For local patients, information was cross-checked with EPR, MDSAS or local database systems, where possible. This could not be repeated for national data. This issue was most noticeable for ‘geographical location’ of burn injury in which contradictory, absent and erroneous data were frequently identified. In the Greater Manchester paediatric data, local data collection systems prior to 2016 were limited, resulting in likely incomplete information and omission of patients from the years 2010–2015. This may influence the trend increase in paediatric sunburn injuries observed locally and prevented regression analysis over the 10-year study period. This trend however was still observed in the national data.

## 5. Conclusions

This 10-year retrospective review of sunburn injuries finds evidence of an increasing presentation of sunburn injuries presenting to the adult and paediatric burn centres in Greater Manchester. Although such injuries were typically superficial and small in size, sunburns appear to be increasing. This pattern was also observed in national data from England and Wales recorded in the iBID database. These findings support the need for further patient engagement and awareness activities to reduce risk of sunburn. This is of greatest value in paediatric and younger adult populations, to reduce risk of long-term complications including melanoma and non-melanomatous skin cancer.

## Figures and Tables

**Table 1:** Demographics and characteristics of all sunburn injuries presenting to the combined Manchester Burns Services 2010–2019 (incl.). ND—not documented, TBSA—total body surface area, SPT—superficial partial thickness, DPT—deep partial thickness, FT—full thickness. * mean/median calculated from patients with documented burn of greater than 0% for a given depth. ⤈ some patients had no documented depth of burn, hence were not included in analysis by depth.

	Adult	Paediatric
Total Patients, *n*	228	131
Male	118	88 (67.2%)
Female	110	43 (32.8%)
Age (years)	16–19—29 (12.7%)	0–1—21 (16.0%)
Patients [*n*, (% of total)]	20–24—37 (16.2%)	2–4—16 (12.2%)
	25–34—52 (22.8%)	6–10—27 (20.6%)
	35–44—29 (12.7%)	11–15—75 (15.1%)
	45–54—37 (16.2%)	
	55–64—25 (11.0%)	
	65–74—11 (4.8%)	
	75+—8 (3.5%)	
Mean [years, (SD, range)]	32.0 (17.3, 16–83)	8.9 (5.0, 0–15)
Total Burn TBSA [Patients, *n* (% total)]		
0	33 (14.5%)	0
0–0.9	68 (29.8%)	42 (32.1%)
1–4	96 (31.6%)	76 (58.05)
5–9	23 (10.1%)	13 (9.9%)
10–14	6 (2.6%)	0
ND	2 (0.9%)	0
Mean [%, (SD)]	2.00 (2.53)	2.12 (1.85)
Median (%)	1	1.5
Range (%)	0–14	0–9
Burn Depth		
Superficial/SPT		
Patients [*n*, (% of total)]	185 ^⤈^ (81.1%)	131 (100%)
Mean TBSA * [%, (SD)]	2.22 (2.37)	2.09 (1.84)
Median TBSA * (%)	1.5	1.5
Range	0–14	0–9
DPT/FT		
Patients [*n*, (% of total)]	11 ^⤈^ (4.8%)	1 (0.8%)
Mean TBSA * [%, (SD)]	0.44 (0.50)	N/A
Median TBSA * (%)	0.1	N/A
Range	0–1.5	N/A
